# The Registered Practical Nurse (RPN) Role in an Academic Acute Care Hospital: A Mixed Method Study of the Barriers and Facilitators to Practice

**DOI:** 10.1155/2024/7309242

**Published:** 2024-07-25

**Authors:** Natalie Weiser, Melanie Dissanayake, Cecilia Santiago, Fiona Harrington, Nichelle Benny Gerard, Sarah Dimmock, Sonya Canzian, Jane Topolovec-Vranic

**Affiliations:** St. Michael's Hospital Unity Health Toronto, 30 Bond St, Toronto M5B 1W8, ON, Canada

## Abstract

**Background:**

Registered Practical Nurses (RPNs) are considered a critical component of high functioning nursing and interprofessional care teams. Therefore, it is important to ensure that RPNs feel valued within their roles within acute care settings. High acute care demands in tandem with unsupported workplace environments can lead to increased levels of job dissatisfaction, burnout, and ultimately impact retention. Identifying and examining the barriers and facilitators that enable RPNs to be optimally equipped within acute care are critical towards ensuring success in their role. In this study, we explore the experiences of RPNs and perspectives of nurse leaders on RPN integration into an acute care setting.

**Methods:**

A mixed method study among RPNs (*n* = 10) and nurse leaders (*n* = 10) was conducted. This included administration of the Assessment for Collaborative Environments (ACE-15) tool to measure interprofessional integration, collaboration, and teamwork. Semi-structured interviews were also held with all participants to explore both the lived experiences of RPNs in the acute care environment and the perspectives of nurse leaders who had supported the onboarding and integration of RPNs.

**Results:**

Our inductive content analysis identified 5 themes: preintegration process, nursing team dynamics, RPN role clarity, challenges to RPN integration, and benefits to RPN integration. ACE-15 data showed no significant differences in the level of teamness and internal disagreement between RPNs and nurse leaders (t (17) = 0.37 and *p* = 4.60). RPNs reporting a higher level of teamness described a more positive integration experience than those who reported a lower level of teamness.

**Conclusion:**

The integration of a new role to existing teams brings both benefits and challenges which are experienced uniquely by RPNs and nurse leaders. Nurse leaders can utilize findings of this study to better prepare their staff and units for the integration of new roles into their models of care.

## 1. Introduction

A shortage in nursing capacity within acute care settings continues to burden the Ontario healthcare system [1]. As a result, nurses are experiencing higher levels of burnout, while patients are experiencing compromised levels of quality patient care [2]. To alleviate burdens due to nursing staffing shortages, new nursing care delivery models are being implemented [3, 4]. Such models include the reintroduction of Registered Practical Nurses (RPNs) into acute care [4].

In Ontario, Canada, nursing is one profession with multiple categories which include Registered Practical Nurse (RPN) and Registered Nurse (RN). The main distinction between RNs and RPNs is foundational education. While RNs and RPNs acquire similar nursing knowledge, RNs study for a longer period of time, allowing for a greater depth and breadth of foundational knowledge [5]. In Ontario, a two-year diploma is required for RPNs and the nursing curriculum consists of courses such as basic human anatomy, practical nursing theory, and practical skill labs. RNs usually complete a four-year post-secondary university nursing program. Although there are significant areas of overlap for the two categories of nurses, differences in responsibility can be identified in terms of the depth, breadth, and scope of involvement with patient care [6]. RPNs are employed in various settings including long-term care, hospitals, and community settings.

The additional educational requirements over the years have enabled RPNs to increase skills in clinical training, thereby expanding overall scopes of practice [7]. Furthermore, the literature has identified that RPNs are considered a critical component of high functioning nursing and interprofessional care teams [3, 8]. Therefore, it is important to ensure that RPNs feel valued within their roles within acute care settings.

High acute care demands in tandem with unsupported workplace environments can lead to increased levels of job dissatisfaction and burnout, and this ultimately impacts retention [9]. Identifying and examining contributing factors that enable RPNs to be optimally equipped within acute care are critical towards ensuring success in this role.

Over time, hospitals try to respond to changing workforce demand environments by adjusting their models of care. Our acute care centre is one such example. In the 1990s, our organization was an RN and RPN staffed hospital. The RPN role was more task-based at this time and RPNs worked under the supervision of an RN. During this time, the organization phased out the RPN role in preference of an exclusively RN staff. In response to changing demands including staff shortages, the RPN position has been reintroduced. While the RPN role has been reintroduced, it is important to be mindful that the RPN role has evolved over the past 30 years, with much more autonomy in practice. Furthermore, role evolution has resulted in overlapping scopes of practice with the RN role. Therefore, it is essential to understand the challenges and opportunities presented with the integration of the RPN role into the acute care setting.

Our study explores the experiences of the RPNs as they were reintegrated into our acute care setting. In particular, we evaluated how RPNs adjusted to work within an acute care setting that was previously all RN staffed through exploring the barriers and facilitators to practice. We also explored the perspectives of Nurse Leaders (NLs) including Clinical Leader Managers (CLMs), Clinical Educator-Nursing (CE-Ns), and Charge Nurses (CNs) in order to identify current and potential strategies which would best support the transition of RPNs to the acute care environment. Moreover, our study aimed to identify what strategies increased confidence and readiness to care for various patient assignments across differing units.Findings from the perspectives of RPNs and NLs were synthesized in order to present a fulsome representation of the RPN integration experience.

## 2. Methods

### 2.1. Setting

This study took place at an academic health science centre in Toronto, Canada. Our organization is a 450 bed centre which provides primary, tertiary, and quaternary care services. The clinical workforce is comprised of approximately 1914 nurses (1760 RNs and 154 RPNs) and 950 other health discipline clinicians.

### 2.2. Study Design

The study employed a mixed method approach utilizing both quantitative and qualitative methodologies. Quantitative data were collected using the Assessment for Collaborative Environments (ACE-15) tool as well as a demographic survey to describe participants' work history. The demographic survey included clinical role, length of time on the unit, employment status, and the timing of typical shifts worked (i.e., weekday and weekend).

The ACE-15 is a 15-item measure with each item scored on a 4-point Likert scale ranging from 1 (strongly disagree) to 4 (strongly agree). Example items include “Team members appreciate each other's roles and expertise” and “All voices on the team are heard and valued” [10]. The total item scale runs from a reported low level of integration of 15 to a high level of integration of 60. The higher the score, the higher the perception of interprofessional integration, collaboration, and teamwork. An inductive qualitative approach using semi-structured interviews was utilized to explore RPN and NL perspectives on the integration of the RPN role at our organization. In particular, interpretative phenomenological analysis (IPA) was the framework used to analyze qualitative data. IPA employs a phenomenological, hermeneutic, and idiographic approach together within one methodology, thereby being the most suitable to approach the phenomenon of interest [11].

### 2.3. Participants

RPNs were eligible to participate in this study if at the time of the study they were employed as an RPN at our organization and had 3 months or more experience as an RPN at the study site. NLs were eligible to take part in the study if at the time of the study they were employed at the organization and responsible for the onboarding and nursing orientation of RPNs.

### 2.4. Procedures

Ethical approval of this study was received from the Research Ethics Board of the hospital. Eligible participants were recruited through an emailed study invitation letter from CLMs and CE-Ns who have RPNs on their units. The study was also advertised through the hospital's broader communication channels and biweekly newsletters as well as existing mailing lists. Potential participants were provided with the study team's contact information and were instructed to contact the study coordinator for additional information. The study coordinator reviewed the study with interested participants, obtained consent, and sent participants an online survey link which had both a demographic survey and the ACE-15 measure. Following completion of the survey and ACE-15, the research coordinator scheduled an in-depth interview with participants.

### 2.5. Interviews

All participants took part in semi-structured individual interviews which ran between 30 and 60 minutes. Interviews took place using Zoom, and audio recordings were extracted for transcription. At the start of all interviews, participants provided verbal consent to ensure willingness to continue taking part in the study. Interviews were scheduled at a time that was convenient to participants. A study team member (N.W.) with expertise in qualitative interviewing completed interviews with all participants.

Two separate semi-structured interview guides (RPN and NL versions) were developed (Appendix A). The RPN interview focused on onboarding and orientation to the hospital and integration to particular units, preparedness for the RPN role, supports (people and resources) in daily work activities, and challenges in the RPN role. The NL participants were asked about the onboarding and orientation of RPNs, the impact of RPN introduction on teamwork dynamics, and the benefits and barriers to RPN integration. As the interviews were semi-structured, participants had the flexibility to discuss additional topics and experiences which they deemed significant to the study topic. Data collection ended when the research team collectively agreed that thematic saturation was reached.

### 2.6. Data Analysis

Descriptive statistical analysis was performed. Proportions were generated for categorical data. Means, standard deviations, medians, and range were generated for each item in the ACE-15 measure from all respondents. Means, standard deviations, medians, and the range were summed to create an overall score. A two-tailed unpaired *t*-test was used to determine if there was a significant difference in the overall score of teamness between RPNs and NLs.

All interviews were audio recorded using Zoom and transcribed verbatim. Two members (N.W. and M.D.) of the study team individually completed an inductive content analysis beginning with a line by line review of all the transcripts in order to develop a comprehensive understanding of the data and ensure the subsequent coding trees were created with fidelity to the data (Appendix B). RPN and NL interviews were treated as separate datasets. N.W. and M.D. together generated a set of preliminary codes through open coding [12]. To develop reliability and trustworthiness, the transcripts were coded individually by N.W. and M.D. and emerging codes were compared and reviewed until consensus was achieved. Following this, the initial coding tree was revised and previously coded transcripts were recoded as needed to incorporate newer codes. Finalized codes were discussed with all research team members in order to integrate RPN and NL themes in the final analysis.

## 3. Results and Discussion

### 3.1. Participant Demographics/Characteristics of Study Participants

16 RPNs responded with interest to either a study invitation letter or hospital wide communications advertising the study. 4 did not reply to a response e-mail, outlining study participation requirements, 2 did not complete the interview due to scheduling difficulties, and 10 completed the interview. 10 NLs responded to the study invitation, and 10 completed interviews. In both groups, 70% of the participants (*N* = 20, *χ*^2^ (1) = 0.95, and *p* > 0.05) have been working on the unit for under a year. 65% of the participants (*N* = 20, *χ*^2^ (1) = 1.98, and *p* > 0.05) have been in the nursing profession for more than 5 years. Majority of the participants (80%, *N* = 16, *χ*^2^ (1) = 1.25, and *p* > 0.05) worked full time compared to part time. Majority of the RPNs (90%, *N* = 9) worked rotating shifts, whereas majority of the NLs (80%, *N* = 8) worked day shifts. A chi-square test of independence showed that there was a significant relationship between day and rotating shifts (*χ*^2^ (1) = 9.90 and *p* < 0.05).

### 3.2. ACE-15

The ACE-15 tool is an assessment of the quality of interprofessional teamwork in clinical sites. This measure is a 15-item, self-report survey appropriate for a broad array of health professionals working in a variety of clinical sites. Scores from the NLs' responses ranged from 37 to 60 with a mean of 48.6 and a standard deviation of 8.6, whereas the scores from the RPN responses ranged from 31 to 60 with a mean of 46.9 and a standard deviation of 11.1. A higher mean score and a lower standard deviation indicate more teamness and less internal disagreement. All of the RPNs and 9 of the NLs completed the ACE-15 (95% response rate). There was no significant difference in the level of teamness when comparing the NLs with the RPNs (*t* (17) = 0.37 and *p*=4.60).

**Figure 1:**
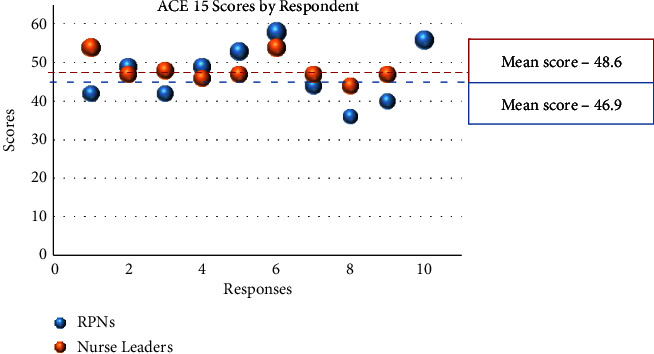
ACE-15 scores.

Figure 1 shows these results pictorially.

### 3.3. Qualitative Themes

Our inductive content analysis identified 5 themes. 4 themes ran across the two participant groups including preintegration process, nursing team dynamics, RPN role clarity, and challenges to RPN integration. One theme unique to the NLs was benefits to RPN integration.

#### 3.3.1. Preintegration Process

RPNs and NLs both described the process of preparing individual teams and/or units for the integration of RPNs. The extent to which established teams were receptive to the introduction of RPNs onto their team was shaped by the education and preparation from NLs as well as the extent to which RNs had prior experience working with RPNs.

Both groups explained that there were RN concerns around workload, patient safety, job security, and general uncertainty about the new team model. Participants also discussed how pre-existing beliefs and assumptions about RPNs needed to be addressed and the addition of RPNs was framed as an opportunity to enhance the strengths of the existing team.

*I think it was fortunate for me having worked with RPNs in the past, like helping the roll out of RPNs on the unit it came in handy for sure. Because I think like part of the stressful part about introducing RPNs is there are a lot of people who've never worked with RPNs before and they didn't know, you know, what it would look like? What does that mean to me and my workload, or to the patients and all that. So, having that experience, made it really helpful. (NL 019)*

*A lot of them [RNs] were very hesitant for us to be integrated even into the area that we were integrated into originally, because they had no knowledge about what our scope was, and instead of just educating themselves on what our scope is, made presumptions about what we could and couldn't do. (RPN 004)*

Another NL suggests that nurses who have worked with RPNs in the past should speak about their collaboration experiences with their current team. This would be helpful to alleviate or address some of the concerns of existing team members and provide a sense of what the new team model will look like in terms of working with the patient population on the particular unit and how the new roles will inform one another.

*I think first talking to the RN team, if they're not used to working with RPNs, and identifying any challenges that they foresee and then addressing those challenges before integrating the RPNs would help a lot, especially with the culture, if it's going to be a huge culture shift. You don't want to integrate RPNs when RNs are not being receptive. So addressing those barriers before integrating the RPNs into the unit, I think would be very beneficial. (NL 007)*

Many participants addressed the culture of the unit as a salient aspect of the preintegration landscape. One of the NLs describes her unit as being fairly new and therefore lacking an established culture or history of a particular nursing model. As such, with the onboarding of RPNs, there was not a significant change to nursing practice as the unit was still building their processes and procedures.

One NL explained the need to educate the nursing team and build relationships when there is a change and a new blending of team members:

*⋯you can't introduce a new role without someone else feeling like, who is this person? why are they here? what does this mean for me? And honestly, like, I had one nurse who actually said that, like, what does this mean, for me? So having people to talk to about that, and their biggest fears, their, you know, fantasies of what might happen, I think is critical to success when you're changing skill mix on a team. (NL 010)*

NLs also noted that prior to the RPN integration, there was corporate communication, program level discussion, and unit specific education about the value of the RPN role, the RPN scope of practice, and how RPNs would fit into the existing team.

#### 3.3.2. Nursing Team Dynamics

Nursing team dynamics emerged as a strong theme in the interviews as both RPNs and NL described the nature of the relationships between RPNs and RNs once RPNs were brought into the hospital. Some CE-Ns and CLMs discussed tension early on as RPNs joined their teams. The RPNs also explained that there was initially some resistance when they joined their teams. As one RPN reflects,

*There did seem to feel like a little bit of a divide, I don't know if that was just like me just overthinking things. But after a couple of weeks, in really getting to know the other staff, I could kind of tell that they didn't really care as long as the patients were kept safe. (RPN 009)*

Another RPN describes that when they first came to the hospital, there were concerns about whether they could be trusted as capable members of the nursing team. An important component of integrating onto the team was patience as trust between RPNs and RNs was established.

*I think when they when we [RPNs] first came in, they [RNs] felt like they couldn't trust us. I kept telling myself we are new to each other and it takes years. Just be patient. (RPN 003)*

A NL describes the shift in RN feelings towards RPNs postintegration:

*I believe that especially a couple of my naysayers, they've come around. I think it's because we had to prove it, they had to live it, they had to see that, in fact, this [RPN integration] wasn't negatively impacting their assignments, and that these people [RPNs] were great team players, and that care being provided for patients remains the same. (NL 015)*

Peer support between RPNs on a team was highlighted as a strategy to mitigate the tensions felt during the early days of integration. As one RPN explains, “a lot of the times, RPNs have to support RPNs as much as we can” (RPN 004).

Participants explained that over time there was a positive shift in the dynamics of the teams. The teams were described as being more cohesive and supportive. Many explained that they felt they were a nursing team as opposed to “RPN vs. RN.” One RPN notes that she felt very supported by her team members and this gave her confidence to ask questions as she was learning the new role.

*I was always supported when it came to my shadowing shifts. I always had somebody with me and anytime I had any type of questions, I would just full on ask them. I never felt afraid to ask any type of questions when it came to care. (RPN 008)*

#### 3.3.3. RPN Role Clarity

Participants explained that the role of the RPN as a unique designation with separate responsibilities from RNs was not always clear. One RPN explains, “RPN and RN care is not black and white, it depends on the care, patient, and staff who are on” (RPN 005). Another RPN states, “they limit what we can take on as RPNs. And sometimes it feels like there's a very blurred line” (RPN 009). The lack of role clarity was especially prevalent in units where there is less acuity in the patient population. As described in the interviews, RPNs are meant to take on patients who are more stable and predictable, while RNs accept patients who are less stable and less predictable. As such, more patient acuity in a unit suggests more differentiation between RPN and RN roles.

*Generally, I do know that what is supposed to happen is that the patients who are more acutely ill, and whose status changes quicker, those patients are technically supposed to be given to the RNs and the more stable patients are supposed to be given to the RPNs. I don't believe in my personal opinion that is very true on this particular unit. I think that on this particular unit that all of the nurses are given equal assignments. (RPN 008)*

*So usually, they would like to assign patients with predictable outcomes to RPNs, anything that they're concerned where a patient may change, and it will require a lot more critical thinking, analysis work from an RPN, that's when you would choose to give the patient to an RN. But at the same time, it doesn't negate what an RPN can do, they could still have a more critical patient, but just making sure that there is an RN available for guidance in case of any kind of concerns. And then if it was just too beyond their scope, then you transition the care to the RN. So, I think on our unit, in general, since our patients are typically pretty predictable outcomes, there isn't much difference in how we are assigning our patients for an RN versus an RPN. (NL 012)*

Some NLs also described that because the tasks of RPNs and RNs are often very similar, there are scenarios in which healthcare professionals or patients and families will not know whether a nurse is an RPN or an RN.

*You know, there's many things that the RPN can now do that they once couldn't do, but to actually see the tangible difference in regards to tasks it's very hard actually, to tell the difference between an RN and RPN. And if you ask a lot of our physicians, I don't think that they actually know the difference between the two. They see all of our nurses as quote un quote, nurses with different levels of experience. (NL 001)*

One of the NLs predicts that “going forward for the future, I really see that the RPN and RN role is blending, like, I don't see the clarity between them” (NL 012). Another NL however cautions that blurring the line between various healthcare professional roles is not desirable and can compromise patient safety:

*I think it's absolutely critical that there are clear identities and roles. You know, you have a special designation with a college of nurses, it should be clear to everyone, whether you're a nurse, an RPN, a social worker, an OT. Patients should be able to ask, family members should be able to ask, and I think just saying we're all just nurses, that just kind of minimizes it and from a safety perspective it's terrible. I don't like that idea that we're all one at all. (NL 010)*

#### 3.3.4. Challenges to RPN Integration

The challenges to the RPN integration were largely related to the setting being an RN only environment and a lack of understanding about what the RPN role would contribute to the established team composition.

*And so it came with a lot of either positive or negative preconceived notions about what an RPN could bring to the table. I found that was probably one of the toughest challenges that we encountered was getting past people's, like myths, almost it was like it was things that people had heard, it's not that it was rooted in any sort of evidence. (NL 001)*

One RPN explains how acceptance onto her unit was made difficult by team members who were not as open to the change in the nursing care delivery model:

*If you were to ask me to give advice to someone that is onboarding, I would say, don't really take everything personally. Because it really depends on the personality of who you're dealing with. And that just could be who they are. And kind of just, they're adjusting to you joining their unit as much as you are adjusting to being on the unit. Because it's like, that's their home. And that's what they're comfortable with. And some people don't like change. (RPN 009)*

NLs also described structural challenges such as compensation discrepancies between nursing roles. Because RPN and RN tasks overlap in many respects, some NLs note that there is RPN dissatisfaction about pay inconsistency. Moreover, the lack of distinct nursing responsibilities between RPNs and RNs on some units raises questions about the distinct nature of the RN position:

*The challenge I'm getting faced with now is, as I am rolling it out, I'm hearing two different things; what is the RN identity? What is our unique role in this hospital? And I guess, yeah, the use of the unique role of the RN has come up and then from an RPN point of view, I've heard such comments as why are we getting paid so much less and doing the exact same work? (NL 014)*

Another NL elaborates that there is “tension amongst the team more so for the RPNs. Because the RPNs feel like, why am I getting paid like half your wage, when I'm doing literally the exact same job as you” (NL 017).

RPNs discussed the emotional impact of their integration as they highlighted feelings of being undervalued on their teams. As some RPNs were introduced to the organization during the early phases of the COVID-19 pandemic, some were first brought on as screeners in the emergency department. This was described as a source of frustration because RPNs felt they were not given an opportunity to utilize their nursing skill set.

#### 3.3.5. Benefits of RPN Integration

NLs explained the benefits of integrating RPNs to the acute care setting from a staffing perspective. Some noted that RPNs delivered excellent patient care, they work as collaborative members of the team, and they are valued for their feedback about practice on the units. Moreover, RPNs were described as being experts in their particular fields who can support the nursing team as some RPNs came to the acute setting with decades of experience as internationally trained nurses or with experience from other settings such as complex care or long-term care.

Many interviews described the context of the COVID-19 pandemic which compounded prevailing staffing challenges. The addition of RPNs was beneficial as they were able to provide more hours of nursing services for patients.

*They [RPNs] kind of provided us some flexibility in our staffing model. I think the pandemic too has been an interesting challenge. I guess in regards to that, like, I think the amount of registered nurses' vacancies that we've had on our particular unit, in the past, if you couldn't get a registered nurse that position would sit vacant. And so I guess too maybe it depends on the CLM but we were really able to increase our staffing capacity by hiring RPNs. (NL 001)*

*Whenever I brought in an RPN, there was a relief, yes, another full time [nurse] onboarded. I felt like people really welcomed, they wanted the expansion of the team. (NL 006)*

The improvement of quality of patient care was also discussed as a benefit to RPN integration. By opening the workforce to RPNs, there is a wider pool of applicants from which nurses are hired. A NL states, “I think it's improved the quality of care in that we are getting the correct person and not someone based on a limited qualification. So we're getting more diversity in nursing and nursing knowledge” (NL 014).

### 3.4. Relationship between ACE-15 and Qualitative Themes

We aimed to assess whether self-reported levels of teamness on the unit were reflected in the respondent interviews. Accordingly, we re-explored the transcripts from RPN participants with the highest and lowest ACE-15 scores. The RPNs with the highest ACE-15 scores explained in their interviews that they felt very supported by their teams:

*At first everyone was kind of touchy of like, what we can do and what can't we do? And now like we're more open, and we're more talkative with management and the unit leader and educator. (RPN 002)*

*I felt so supported my very first solo day, I think all of my buddy nurses, like, two nurses above me, when it came to the rooms and then two nurses behind me and the charge nurse, everybody knew that I was going solo and they kept checking in all the time. They're like, how are you doing? Do you need any help with this? I felt very, very supported. (RPN 008)*

RPNs with the lowest ACE-15 scores noted feeling “underutilized” and distrusted by RNs on their teams:

*Even to this day you can sometimes see where the RNs are hesitant to have RPNs, or just like that trust factor is very, like not, it's not automatic. Whereas a new grad student that just got out of an RN program, they have more trust than me, where I've been working as RPN for five years and on this floor for a year and a half now, but a new grad that just started last month is going to have more trust. (RPN 004)*

Overall in this subanalysis, RPNs reporting a higher level of teamness described a more positive integration experience than those who reported a lower level of teamness.

## 4. Discussion

The integration of a new role to existing teams brings both benefits and challenges which are experienced uniquely by RPNs and NLs. Specifically, our in-depth study identified the following themes common to both groups: preintegration process, nursing team dynamics, RPN role clarity, and challenges to RPN integration. One theme unique to the NLs was benefits to RPN integration. Moreover, our secondary analysis suggested that RPNs working on units with high levels of teamness generally had a more positive experience than those with low levels of teamness.

There are a variety of ways to enhance teamness and address gaps or challenges in team dynamics. Education or learning and development portfolios at healthcare organizations could develop and implement teamwork educational workshops and programs for fostering both inter- and intraprofessional collaboration [13]. Other examples include setting up charters for how teams will work together, protecting time in meetings for discussion about roles and scopes of practice, shadowing different professions to enhance role clarity and individual courses through learning centres. Teams might also employ validated tools such as the ACE-15 to assess levels of teamness on their units and address gaps either prior to or as part of the integration of new roles. Increasing teamwork among patient care teams is valuable as it can positively influence both job satisfaction and patient care [14].

Consensus among teams about roles and scopes of practice are also essential to a successful implementation of new nursing roles. Teams must have role clarity and understand how the various nursing positions fit into the wider team in order to have collaborative relationships among one another [15]. However, this is an ongoing process as factors such as evolving patient needs, interprofessional team models, and other healthcare system changes require constant monitoring and assessment of role clarity and scope of practice for all professions, including RPNs [16].

This study had both strengths and limitations. Our study examined two sets of nursing perspectives, utilized a mixed method approach, and was conducted at an organization that is early in its RPN integration process. Because RPNs are very new to the site, the findings are near real time. Finally, we included an RPN in the data coding process which allowed for a rich and nuanced analysis which was grounded in the RPN's clinical experiences. A limitation of the study is that NL participants may have been more supportive of RPNs than those who chose not to participate in the study. Conversely, RPNs who chose to take part in the study may have been those with more negative experiences and a self-selection bias may have been at play. A second limitation is that we did not include Registered Nurses (RNs) as participants other than those who are both RNs and NLs. The RN perspective would be valuable to an understanding of the nursing dynamics theme.

A strong theme that emerged from NLs was the necessity of preintegration activities that would help a unit succeed in the introduction of the RPN role. In particular, dedicating time to educating existing team members about the scope of RPNs' practice was explained as vital to the success of the integration process. In addition, a focus on the value RPNs bring to the team is important to highlight especially in circumstances in which team members are new to working with RPNs. Other NLs discussed having conversations with teams about RPN integration and the shifting dynamics of a new skill mix on the team. In this way, current team members would be educated and prepared for collaborating with RPNs.

The theme of role clarity suggests the value of developing unit-specific guidelines for tasks assigned to RNs and RPNs. Role clarity would allow for a common understanding of one another's roles for the entire interprofessional team. A shift assignment toolkit, for example, can enhance role clarity and collaboration with respect to patient assignments. A standardized resource on shift assignment can also help healthcare organizations diminish role ambiguity among newly introduced RPNs in acute care settings.

## 5. Conclusion

The integration of a new role to existing teams brings both benefits and challenges which are experienced uniquely by RPNs and NLs. NLs can utilize findings of this study to better prepare their staff and units for the integration of RPNs but also any shifts or changes in their models of care. Our findings could be used to improve RPNs' integration in acute care, optimize the professional nursing environment to allow RPNs to practice to their full scope, and enhance the quality of patient care. Future studies can explore other interprofessional team members' perspectives on RPN integration to provide a more fulsome picture of the integration process. For example, the RN perspective would be valuable in further exploring the nursing dynamics theme. Additional work could also evaluate strategies to help address some of the themes identified above by increasing levels of teamness to boost team dynamics. Finally, how these strategies affect long-term outcomes such as burnout and nursing retention remains to be explored.

## Data Availability

The data that support the findings of this study are available upon reasonable request from the authors and with permission from the ethical bodies.

## Supplementary Materials

Supplementary Materials Appendix A shows two semi-structured interview guides (one for Registered Practical Nurses and one for Nurse Leaders). Appendix B shows the codebooks developed and utilized during data analysis for both Registered Practical Nurses and Nurse Leaders.
