# Supplementary material for: The Registered Practical Nurse (RPN) Role in an Academic Acute Care Hospital: A Mixed Method Study of the Barriers and Facilitators to Practice
Source: J Nurs Manag. 2024 Jul 25;2024:7309242. doi: 10.1155/2024/7309242 (PMC11919085; doi:10.1155/2024/7309242)
Supplement: Supplementary Materials — Appendix A shows two semi-structured interview guides (one for Registered Practical Nurses and one for Nurse Leaders). Appendix B shows the codebooks developed and utilized during data analysis for both Registered Practical Nurses and Nurse Leaders. [file 7309242.f1.zip › Appendix A_Interview Guide.docx]

**Appendix A: Interview Guides**

1. **Interview Guide - RPNs**
2. Please tell us about your background, education and training, work experience as it pertains to the RPN role.
3. How did onboarding and orientation training prepare you for working as a part of the team in the unit?

Probes:

- - Is there anything additional you would have liked to have included in your orientation?
  - Were there any topics covered in the orientation you:
    1. Found especially useful to your role?
    2. Did not find it useful or relevant to your role?
  - Is there anything you wish you would have learned more about in the orientation?

1. Following orientation, to what extent did you feel prepared for the scope of work the role entailed? Please describe.

Probes:

- Are you able to engage with other team members regarding patient care? For example, regarding the transfer of accountability from RPN to RN and vice versa.

To what extent are you comfortable with the assignments on the unit?

- To what extent do you feel the assignments are flexible and appropriate to your scope of practice in terms of the complexity of patients’ care, your current skill set and knowledge?

1. Could you please describe how your integration into practice was supported on the unit initially and if there any ongoing supports now?

Probes:

- What other supports would you have liked or found valuable?
- Are there any supports you would like to have that are missing? Please describe.

1. Could you describe any people or resources that help you the most in your day to day work on the unit?
2. What are some of the challenges you face related to your role in the unit?

**b.) Interview Guide – Nurse Leaders**

1. Please tell us about your experience in working with RPNs in the past.
2. What information did you receive about the RPN role and scope of practice in acute care prior to their onboarding?
3. What are you looking for in a candidate when hiring a RPN to work in your unit?
4. How many RPNs do you have working in your area?
5. How often, on average, are RPNs reassigned to different tasks within the unit or leave your unit?
6. How did having RPNs on your unit impact teamwork?

Probes:

- - (may use ACE-15 responses to guide): e.g., improved satisfaction, decreased burden, decreased workload, patient care needs being met.

1. What are the benefits you experienced with optimizing RPNs role on your unit? Please describe.

Probes:

- - (may use ACE-15 responses to guide): e.g., RPNs work to the full scope of practice, deliver excellent quality of care and improve patient outcomes.

1. What are some of the challenges or barriers, if any, you faced with integrating RPNs on your unit?

Probes:

- - What suggestions do you have to overcome these challenges?
  - Are there any gaps in terms of expectations or RPNs training in acute care settings?

1. What helps in supporting the integration of RPNs into their role? Can you please describe how RPNs integration into practice was supported on the unit and if there any ongoing supports.
2. What suggestions do you have to improve the onboarding and orientation training program? Please describe.
