# Supplementary material for: The Registered Practical Nurse (RPN) Role in an Academic Acute Care Hospital: A Mixed Method Study of the Barriers and Facilitators to Practice
Source: J Nurs Manag. 2024 Jul 25;2024:7309242. doi: 10.1155/2024/7309242 (PMC11919085; doi:10.1155/2024/7309242)
Supplement: Supplementary Materials — Appendix A shows two semi-structured interview guides (one for Registered Practical Nurses and one for Nurse Leaders). Appendix B shows the codebooks developed and utilized during data analysis for both Registered Practical Nurses and Nurse Leaders. [file 7309242.f1.zip › Appendix B_Codebook.docx]

**Appendix B Code Books**

**Code Book for RPNs:**

| 1. Defining the RPN Role - Role Clarity |
| --- |
| 2. Team Dynamics/Relationships/Teamness |
| 3. Role Confidence |
| 4. Pre-Integration Process/Information |
| 5. Role Stigma |
| 6. Future Scope of RPN Role |
| 7. Context of Acute Care Hospital |
| 8. Emotions |

**Code Book for Nurse Leaders (NLs):**

| 1. Pre-Integration Information/Education |
| --- |
| 2. Perception of RPNs/Receptiveness of Team to RPNs |
| 3. Context (staffing shortages, Covid-19 pandemic, culture of unit (established unit) |
| 4. Ideal RPN Skill Set = ideal qualities/skills when looking for RPN |
| 5. Patient Assignment |
| 6. Role Clarity/Scope of Practice |
| 7. Benefits of bringing RPNs on to team |
| 8. Challenges/Barriers to bringing RPNs on to team |
| 9. Confidence and Performance |
| 10. Future Skill Development/Role Expansion |
| 11. Professional Development and Education |

**Key Themes Running Across 2 Participant Groups (RPN and NLs)**

| 1. Pre-Integration Process |
| --- |
| 2. Nursing Team Dynamics |
| 3. RPN Role Clarity |
| 4. Challenges to RPN Integration |
